# Supplementary material for: Long term 5G network traffic forecasting via modeling non-stationarity with deep learning
Source: Commun Eng. 2023 Jun 6;2:33. doi: 10.1038/s44172-023-00081-4 (PMC10955930; doi:10.1038/s44172-023-00081-4)
Supplement: Supplementary file 2 — supplementary information [file 44172_2023_81_MOESM2_ESM.pdf]

# Supplementary Information

## Supplementary Table 1

Table S1 presents experimental results with statistical analysis of the NPT–1 dataset. The prediction spans of {96, 288, 672, 1344, 2880} are aligned with {1, 3, 7, 14, 30} days, respectively. The average error (AE) indicates the mean errors of different prediction horizons, and the degradation rate (DR) in Table S1 indicates the daily increase rate of errors $\mathrm{dMSE}_1^{30}$ and $\mathrm{dMAE}_1^{30}$ from 1 to 30 days. The best predictive performance over the comparison is shown in bold.

Table S1: Time-series forecasting results on the 5G traffic network dataset.

| Models | | Diviner | | Autoformer | | Informer | | Transformer | | ARIMA | | Prophet | | NBeats | | LSTMa | |
|---|---|---|---|---|---|---|---|---|---|---|---|---|---|---|---|---|---|
| Metric | | MSE | MAE | MSE | MAE | MSE | MAE | MSE | MAE | MSE | MAE | MSE | MAE | MSE | MAE | MSE | MAE |
| *NPT-1* | 96 | **0.256** | **0.340** | 0.456 | 0.511 | 0.264 | 0.349 | 0.259 | 0.333 | 0.401 | 0.487 | 0.456 | 0.531 | 0.491 | 0.509 | 1.778 | 1.092 |
| | 288 | **0.277** | **0.379** | 0.431 | 0.499 | 0.611 | 0.590 | 0.376 | 0.445 | 0.498 | 0.552 | 0.430 | 0.517 | 0.624 | 0.694 | 1.838 | 1.114 |
| | 672 | **0.263** | **0.367** | 0.446 | 0.522 | 1.680 | 0.885 | 0.365 | 0.437 | 0.754 | 0.674 | 0.437 | 0.520 | 0.680 | 0.615 | 1.786 | 1.103 |
| | 1344 | **0.275** | **0.367** | 0.400 | 0.467 | 1.307 | 0.923 | 0.448 | 0.462 | 1.558 | 0.914 | 0.459 | 0.532 | 0.883 | 0.692 | 1.843 | 1.115 |
| | 2880 | **0.318** | **0.390** | 0.674 | 0.629 | 1.590 | 1.050 | 0.811 | 0.652 | 5.522 | 1.632 | 0.550 | 0.576 | 1.257 | 0.844 | 2.251 | 1.221 |
| AE | | **0.277** | **0.368** | 0.481 | 0.525 | 1.090 | 0.759 | 0.451 | 0.465 | 1.746 | 0.851 | 0.466 | 0.535 | 0.787 | 0.670 | 1.899 | 1.129 |
| DR(%) | | 0.750 | 0.474 | 1.356 | 0.718 | 6.387 | 3.871 | 4.014 | 2.343 | 9.464 | 4.258 | 0.648 | 0.280 | 3.294 | 1.759 | 0.816 | 0.385 |

# Supplementary Table 2

Table S2 presents the experimental results of WTH dataset with statistical analysis. The prediction span of {144, 432, 1008, 2016, 4032} is aligned with {1, 3, 7, 14, 28} days. The DR of Table S2 indicates the daily increase rate of errors $\mathrm{dMSE}_1^{28}$ and $\mathrm{dMAE}_1^{28}$ from 1 to 28 days in the WTH case. The best predictive performance over the comparison is shown in bold.

Table S2: Time-series forecasting results on WTH dataset

| Models | | Diviner | | Autoformer | | Informer | | Transformer | | ARIMA | | Prophet | | NBeats | | LSTMa | |
|---|---|---|---|---|---|---|---|---|---|---|---|---|---|---|---|---|---|
| Metric | | MSE | MAE | MSE | MAE | MSE | MAE | MSE | MAE | MSE | MAE | MSE | MAE | MSE | MAE | MSE | MAE |
| *WTH* | 144 | **0.280** | **0.341** | 0.373 | 0.440 | 0.359 | 0.401 | 0.448 | 0.484 | 1.040 | 0.827 | 2.204 | 1.165 | 0.508 | 0.590 | 1.260 | 0.914 |
| | 432 | **0.333** | **0.392** | 0.402 | 0.445 | 0.374 | 0.431 | 0.407 | 0.470 | 1.019 | 0.813 | 2.025 | 1.109 | 0.427 | 0.501 | 1.208 | 0.890 |
| | 1008 | **0.273** | **0.328** | 0.663 | 0.613 | 0.344 | 0.387 | 0.535 | 0.514 | 0.921 | 0.780 | 1.572 | 0.980 | 0.406 | 0.490 | 1.194 | 0.885 |
| | 2016 | **0.233** | **0.306** | 1.857 | 1.019 | 0.367 | 0.417 | 0.367 | 0.417 | 1.022 | 0.840 | 1.246 | 0.833 | 0.757 | 0.635 | 1.220 | 0.900 |
| | 4032 | **0.318** | **0.358** | 1.016 | 0.853 | 1.251 | 0.806 | 0.876 | 0.616 | 0.506 | 0.555 | 1.757 | 1.130 | 0.361 | 0.434 | 1.051 | 0.846 |
| AE | | **0.287** | **0.345** | 0.862 | 0.674 | 0.539 | 0.488 | 0.526 | 0.500 | 0.901 | 0.763 | 1.755 | 1.041 | 0.491 | 0.530 | 1.186 | 0.887 |
| DR(%) | | 0.472 | 0.180 | 3.781 | 2.482 | 4.732 | 2.619 | 2.514 | 0.897 | -2.633 | -1.466 | -0.836 | -0.109 | -1.257 | -1.130 | -0.669 | -0.285 |

# Supplementary Table 3

Table S3 summarizes the experiment results of different granularity ETT datasets including $\mathrm{ETT}m_1$ for 15-minute levels and $\mathrm{ETT}h_1$ for 1-hour levels. The prediction span {24, 48, 168} of $\mathrm{ETT}h_1$ is aligned with the prediction span {96, 288, 672} of $\mathrm{ETT}m_1$, and {1, 3, 7} in days. The granularity change (GC) indicates the relative performance varying when the hour-level granularity turns into the minute-level granularity. The best predictive performance over the comparison is shown in bold.

Table S3: Time-series forecasting results on ETT dataset

| Models | | Diviner | | Autoformer | | Informer | | Transformer | | ARIMA | | Prophet | | NBeats | | LSTMa | |
|---|---|---|---|---|---|---|---|---|---|---|---|---|---|---|---|---|---|
| Metric | | MSE | MAE | MSE | MAE | MSE | MAE | MSE | MAE | MSE | MAE | MSE | MAE | MSE | MAE | MSE | MAE |
| *ETTh1* | 24 | **0.058** | **0.183** | 0.093 | 0.234 | 0.098 | 0.247 | 0.468 | 0.599 | 0.108 | 0.284 | 0.115 | 0.275 | 0.157 | 0.269 | 0.114 | 0.272 |
| | 48 | **0.071** | **0.203** | 0.089 | 0.229 | 0.158 | 0.319 | 0.369 | 0.524 | 0.175 | 0.424 | 0.168 | 0.330 | 0.146 | 0.292 | 0.193 | 0.358 |
| | 168 | **0.199** | **0.262** | 0.148 | 0.280 | 0.183 | 0.346 | 0.478 | 0.618 | 0.396 | 0.504 | 1.224 | 0.763 | 0.494 | 0.536 | 0.236 | 0.392 |
| AE | | **0.082** | **0.216** | 0.110 | 0.247 | 0.146 | 0.304 | 0.438 | 0.580 | 0.226 | 0.404 | 0.502 | 0.456 | 0.265 | 0.365 | 0.181 | 0.340 |
| *ETTm1* | 96 | **0.044** | **0.162** | 0.063 | 0.198 | 0.194 | 0.372 | 0.264 | 0.450 | 0.273 | 0.399 | 0.194 | 0.396 | 1.352 | 1.106 | 0.287 | 0.420 |
| | 288 | **0.078** | **0.209** | 0.096 | 0.245 | 0.401 | 0.554 | 0.230 | 0.410 | 0.462 | 0.558 | 0.452 | 0.574 | 0.628 | 0.621 | 0.524 | 0.584 |
| | 672 | **0.071** | **0.211** | 0.117 | 0.276 | 0.512 | 0.644 | 0.379 | 0.540 | 0.639 | 0.697 | 2.747 | 1.174 | 0.361 | 0.480 | 1.064 | 0.873 |
| AE | | **0.064** | **0.194** | 0.092 | 0.239 | 0.369 | 0.523 | 0.291 | 0.466 | 0.458 | 0.551 | 1.131 | 0.714 | 0.780 | 0.735 | 0.625 | 0.625 |
| GC($\times$100%) | | -0.219 | -0.101 | -0.163 | -0.032 | 1.527 | 0.720 | -0.335 | -0.196 | 1.026 | 0.363 | 1.252 | 0.565 | 1.943 | 1.013 | 2.453 | 0.838 |

# Supplementary Table 4

Table S4 presents the experimental results of the ECL dataset and conducts the analysis of short-term predictions of {7, 14} days / {168, 336} prediction spans and long-term predictions of {30, 40} days / {720, 960} prediction spans separately. The short term average error (STAE) calculates the mean errors of the prediction span of {168, 336} and long term average error (LTAE) calculates the mean errors of the prediction spans of {720, 960}. The DR in Table S4 represents the daily increase rate of errors $\text{dMSE}_7^{40}$ and $\text{dMAE}_7^{40}$ from 7 to 40 days in the ECL case. The best predictive performance over the comparison is shown in bold.

Table S4: Time-series forecasting results on ECL datasets

| Models | | Diviner | | Autoformer | | Informer | | Transformer | | ARIMA | | Prophet | | NBeats | | LSTMa | |
|---|---|---|---|---|---|---|---|---|---|---|---|---|---|---|---|---|---|
| Metric | | MSE | MAE | MSE | MAE | MSE | MAE | MSE | MAE | MSE | MAE | MSE | MAE | MSE | MAE | MSE | MAE |
| *ECL* | 168 | 0.265 | **0.361** | 0.385 | 0.458 | 0.447 | 0.503 | 0.587 | 0.561 | 1.032 | 0.873 | 2.725 | 1.273 | **0.225** | 0.363 | 0.723 | 0.655 |
| | 336 | 0.295 | 0.395 | 0.462 | 0.496 | 0.489 | 0.528 | 0.683 | 0.640 | 1.136 | 0.876 | 2.246 | 3.077 | **0.237** | **0.359** | 1.212 | 0.898 |
| | 720 | **0.303** | **0.409** | 1.349 | 0.907 | 0.540 | 0.571 | 0.482 | 0.527 | 1.251 | 0.933 | 4.243 | 1.415 | 0.367 | 0.482 | 1.511 | 0.966 |
| | 960 | **0.427** | **0.489** | 1.263 | 0.920 | 0.582 | 0.608 | 0.644 | 0.597 | 1.370 | 0.982 | 6.901 | 4.264 | 0.457 | 0.540 | 1.545 | 1.006 |
| DR(%) | | 1.456 | 0.923 | 3.665 | 2.136 | 0.802 | 0.576 | 0.281 | 0.188 | 0.862 | 0.357 | 2.855 | 3.731 | 2.170 | 1.210 | 2.327 | 1.308 |
| STAE | | 0.280 | 0.378 | 0.423 | 0.477 | 0.468 | 0.515 | 0.635 | 0.600 | 1.084 | 0.874 | 2.485 | 2.175 | **0.231** | **0.361** | 0.967 | 0.776 |
| LTAE | | **0.365** | **0.449** | 1.306 | 0.913 | 0.561 | 0.589 | 0.563 | 0.562 | 1.310 | 0.957 | 5.572 | 2.839 | 0.412 | 0.511 | 1.528 | 0.986 |

# Supplementary Table 5

Table S5 presents the experimental results of the Exchange dataset. The prediction span of {10, 20, 30, 60} is aligned with {10, 20, 30, 60} days. The DR here represents the daily increase rate of errors $\text{dMSE}_{10}^{60}, \text{dMAE}_{10}^{60}$ from 10 to 60 days in the Exchange case. The best predictive performance over the comparison is shown in bold.

Table S5: Time-series forecasting results on Exchange dataset

| Models | | Diviner | | Autoformer | | Informer | | Transformer | | ARIMA | | Prophet | | NBeats | | LSTMa | |
|---|---|---|---|---|---|---|---|---|---|---|---|---|---|---|---|---|---|
| Metric | | MSE | MAE | MSE | MAE | MSE | MAE | MSE | MAE | MSE | MAE | MSE | MAE | MSE | MAE | MSE | MAE |
| *Exchange* | 10 | **0.147** | **0.282** | 0.163 | 0.315 | 4.896 | 2.124 | 6.926 | 2.553 | 0.395 | 0.514 | 2.005 | 1.088 | 0.804 | 0.701 | 20.748 | 4.479 |
| | 20 | **0.273** | **0.421** | 0.423 | 0.540 | 6.318 | 2.443 | 6.759 | 2.524 | 0.740 | 0.774 | 2.087 | 1.122 | 1.166 | 0.939 | 21.239 | 4.541 |
| | 30 | **0.399** | **0.506** | 0.857 | 0.799 | 5.388 | 2.253 | 7.307 | 2.635 | 0.874 | 0.869 | 2.172 | 1.155 | 1.521 | 1.105 | 22.021 | 4.626 |
| | 60 | **0.619** | **0.669** | 0.911 | 0.776 | 9.886 | 3.067 | 8.455 | 2.840 | 2.285 | 1.357 | 2.505 | 1.315 | 3.299 | 1.670 | 23.261 | 4.762 |
| AE | | **0.359** | **0.469** | 0.588 | 0.607 | 6.622 | 2.471 | 7.361 | 2.638 | 1.073 | 0.878 | 2.141 | 1.153 | 1.697 | 1.103 | 21.817 | 4.602 |
| DR(%) | | 2.917 | 1.742 | 3.501 | 1.819 | 1.415 | 0.737 | 0.399 | 0.213 | 3.572 | 1.960 | 0.444 | 0.376 | 2.863 | 1.751 | 0.228 | 0.122 |

# Supplementary Table 6

Table S6 shows the experimental results on the Solar dataset. We set the prediction horizon to {1, 2, 5, 6} days, aligned with {144, 288, 720, 864} prediction steps ahead. In Table S6, the average MASE error (AE) indicates the model's comprehensive performances under different prediction horizon settings. The best predictive performance over the comparison is shown in bold. By sorting their comprehensive performances, we have the following results: Diviner >NBeats >Transformer >Autoformer >Informer >LSTMa.

Table S6: Time-series forecasting results on Solar datasets

| Models | | Diviner | Autoformer | Informer | Transformer | ARIMA | Prophet | NBeats | LSTMa |
|---|---|---|---|---|---|---|---|---|---|
| Metric | | MASE | MASE | MASE | MASE | MASE | MASE | MASE | MASE |
| *Solar* | 144 | **7.461** | 11.091 | 8.290 | 11.742 | 31.093 | 29.165 | 8.487 | 18.917 |
| | 288 | **8.355** | 12.035 | 10.007 | 9.289 | 34.362 | 32.236 | 8.988 | 20.327 |
| | 720 | **8.793** | 13.497 | 13.803 | 11.352 | 35.099 | 32.930 | 9.176 | 21.886 |
| | 960 | **7.053** | 14.423 | 21.299 | 11.367 | 32.419 | 30.415 | 8.488 | 20.030 |
| AE | | **7.915** | 12.761 | 13.349 | 10.937 | 33.243 | 31.186 | 8.784 | 20.290 |

# Supplementary Table 7

Table S7 presents the experimental results of the Traffic dataset, and the prediction spans of {168, 336, 720, 960} is aligned with {7, 14, 30, 40} days. The DR in Table S7 presents the daily increase rate of errors $\mathrm{dMSE}_7^{40}, \mathrm{dMAE}_7^{40}$ from 7 to 40 days in the Traffic case. The best predictive performance over the comparison is shown in bold.

Table S7: Time-series forecasting results on Traffic dataset

| Models | | Diviner | | Autoformer | | Informer | | Transformer | | ARIMA | | Prophet | | NBeats | | LSTMa | |
|---|---|---|---|---|---|---|---|---|---|---|---|---|---|---|---|---|---|
| Metric | | MSE | MAE | MSE | MAE | MSE | MAE | MSE | MAE | MSE | MAE | MSE | MAE | MSE | MAE | MSE | MAE |
| *Traffic* | 168 | **0.156** | **0.259** | 0.431 | 0.485 | 1.814 | 1.159 | 0.750 | 0.644 | 1.181 | 0.918 | 1.169 | 0.919 | 0.509 | 0.528 | 1.963 | 1.202 |
| | 336 | **0.158** | **0.261** | 0.437 | 0.477 | 1.799 | 1.153 | 0.629 | 0.573 | 1.182 | 0.919 | 1.169 | 0.918 | 0.517 | 0.529 | 2.007 | 1.214 |
| | 720 | **0.318** | **0.437** | 0.4 | 0.525 | 1.817 | 1.15 | 0.671 | 0.604 | 1.187 | 0.92 | 1.177 | 0.92 | 0.526 | 0.533 | 2.06 | 1.226 |
| | 960 | **0.277** | **0.397** | 0.546 | 0.607 | 1.821 | 1.165 | 1.950 | 1.116 | 1.194 | 0.922 | 1.185 | 0.921 | 0.523 | 0.532 | 1.975 | 1.204 |
| AE | | **0.227** | **0.338** | 0.453 | 0.523 | 1.812 | 1.156 | 1.000 | 0.734 | 1.186 | 0.919 | 1.175 | 0.919 | 0.518 | 0.530 | 2.001 | 1.211 |
| DR(%) | | 1.755 | 1.302 | 0.719 | 0.682 | 0.011 | 0.015 | 2.937 | 1.680 | 0.033 | 0.013 | 0.041 | 0.006 | 0.082 | 0.022 | 0.018 | 0.005 |
