# Supplementary material for: Long term 5G network traffic forecasting via modeling non-stationarity with deep learning
Source: Commun Eng. 2023 Jun 6;2:33. doi: 10.1038/s44172-023-00081-4 (PMC10955930; doi:10.1038/s44172-023-00081-4)
Supplement: Supplementary file 3 — Description of Additional Supplementary Files [file 44172_2023_81_MOESM3_ESM.pdf]

Description of Additional Supplementary File

File name: Supplementary Data 1

Description:

Supplementary Data 1 summarizes all experimental results of the proposed Diviner compared with the time series prediction based baseline models on NPT benchmark.

File name: Supplementary Data 2

Description:

Supplementary Data 2 summarizes all experimental results of the proposed Diviner compared with the time series prediction based baseline models on other real world benchmarks.
